# ‘Cosmetic boob jobs’ or evidence-based breast surgery: an interpretive policy analysis of the rationing of ‘low value’ treatments in the English National Health Service

**DOI:** 10.1186/1472-6963-14-413

**Published:** 2014-09-20

**Authors:** Jill Russell, Deborah Swinglehurst, Trisha Greenhalgh

**Affiliations:** Centre for Primary Care and Public Health, Barts and The London School of Medicine and Dentistry, Queen Mary University of London, Yvonne Carter Building, 58 Turner Street, London, E1 2AB UK

**Keywords:** England, Breast surgery, Health care variation, Rationing, Interpretive policy analysis, Low priority treatments, Value based commissioning

## Abstract

**Background:**

In England the National Health Service (NHS) is not allowed to impose ‘blanket bans’ on treatments, but local commissioners produce lists of ‘low value’ procedures that they will normally not fund. Breast surgery is one example. However, evidence suggests that some breast surgery is clinically effective, with significant health gain. National guidelines indicate the circumstances under which breast surgery should be made available on the NHS, but there is widespread variation in their implementation.

The purpose of this study was to explore the work practices of ‘individual funding request’ (IFR) panels, as they considered ‘one-off’ funding requests for breast surgery; examine how the notion of ‘value’ is dialogically constructed, and how decisions about who is deserving of NHS funding and who is not are accomplished in practice.

**Methods:**

We undertook ethnographic exploration of three IFR panels. We extracted all (22) breast surgery cases considered by these panels from our data set and progressively focused on three case discussions, one from each panel, covering the three main breast procedures.

We undertook a microanalysis of the talk and texts arising from these cases, within a conceptual framework of interpretive policy analysis.

**Results:**

Through an exploration of the symbolic artefacts (language, objects and acts) that are significant carriers of policy meaning, we identified the ways in which IFR panels create their own ‘interpretive communities’, within which deliberations about the funding of breast surgery are differently framed, and local decisions come to be justified. In particular, we demonstrated how each decision was contingent on [a] the evaluative accent given to certain words, [b] the work that documentary objects achieve in foregrounding particular concerns, and [c] the act of categorising. Meaning was constructed dialogically through local interaction and broader socio-cultural discourses about breasts and ‘cosmetic’ surgery.

**Conclusion:**

Despite the appeal of calls to tackle ‘unwarranted variation’ in access to low priority treatments by ensuring uniformity of local guidelines and policies, our findings suggest that ultimately, given the contingent nature of practice, this is likely to remain an illusory policy goal. Our findings challenge the scientistic thinking underpinning mainstream health policy discourse.

## Background

Politicians frequently remind us that there are ‘no blanket bans’ on NHS health care in England [1–3]. Despite such assurances and the absence of national exclusion lists [4, 5], local commissioning organisations^a^ commonly list procedures they will only fund if patients satisfy particular eligibility criteria [6]. These exclusion lists are referred to as ‘low priority lists’ or ‘procedures of limited clinical effectiveness’ [7], terms that are used interchangeably despite subtly different meanings.

The main arguments used to justify disinvestment from interventions of ‘limited clinical effectiveness’ appear to follow the principles of evidence-based medicine. For example, a report from the NHS Right Care project (established to address variation in access to such procedures) states:

*“Most [commissioning organisations] have developed policies on procedures of limited clinical effectiveness, which include criteria to guide treatment and funding decisions. These criteria aim to ensure the NHS commissions interventions which are effective and evidence based and conversely disinvests from interventions where this is not the case”* [8] *.*

Despite the common-sense appeal of such policies, they have prompted considerable debate in the media and between the medical profession, politicians and government. Many of the listed procedures, such as cataract surgery, are of proven clinical value. Critics claim that such lists are primarily a means of reducing expenditure at a time of financial crisis, grounding their counter-arguments in the normative discourse of evidence-based medicine [9, 10].

The NHS Right Care team report explicitly states that ‘low priority’ lists represent more than an assessment of clinical value and that some clinically effective interventions might be deemed ‘low priority’ in terms of their claim on NHS resources [8]. Garner and Littlejohns, reflecting on their experience at the National Institute of Health and Clinical Excellence (NICE) conclude that:

*“many suggestions for total disinvestment are based on a ‘social judgment’ about whether it is appropriate for the NHS to fund the intervention rather than evidence of poor clinical or cost effectiveness”* [11].

The Right Care report recommends a shift in terminology away from ‘procedures of limited clinical effectiveness [or value]’ to ‘value based commissioning’:

*“Terms such as ‘procedures of limited clinical value’ have been identified as a barrier to clinical engagement. There should be a change in terminology to reflect a more holistic approach. ‘Value based’ or ‘effective clinical commissioning’ is proposed as a more acceptable working title”* [8].

The authors of this report do not explain why ‘value based commissioning’ is a more acceptable term (although the words ‘limited’ and ‘low’ have obvious negative connotations), nor how it reflects a more holistic approach. The report makes frequent reference to ‘added value’, ‘better value’, ‘best value’ and ‘high value’ (and occasionally to ‘patients’ values’, indexing a very different meaning to the singular form), but leaves unaddressed the fundamental question of what is meant by ‘value’. Nevertheless, value based commissioning is a term gaining currency in the NHS, with commissioning organisations using it to refer to policy guidance on treatments they will not normally fund.

‘Cosmetic’ surgical procedures are high on the list of treatments that NHS commissioning organisations will not normally fund [12], and are among the commonest requests to local ‘individual funding request’ (IFR) panels^b^, who must consider whether to fund treatment as a special case. NHS commissioning organisations are legally required to have a system that allows patients to argue for a ‘low priority procedure’ or for treatment where no policy (yet) exists [13, 14] to ensure that local commissioners are *“not unreasonably restricting access to treatment for their patients”* [15]. Funding approval rests on the IFR panel deciding a patient is an exception to existing commissioning policies, although as Maybin and Klein note, *“there are no clear and binding guidelines about how ‘exceptionality’ ought to be interpreted or what criteria should be used in decision-making”* [12].

Surprisingly, there are no systematically collected, national data on IFRs, but in one locality ‘plastics’ comprised a quarter of all IFRs and formed the largest single category of requests [16]. Within ‘plastics’, breast surgery (including reduction, augmentation and gynaecomastia correction) accounted for 47% of requests in 2008–9 and 31% in 2009–10 [16].

Breast surgery cases may be trivialised by the media and even by professionals as ‘boob jobs’, and used as an example of what the NHS should not spend limited resources on. Headlines such as *“Why did NHS pay for this woman’s 36DD breasts but refuse to pay £24,000 for an operation so this boy can walk?”* [17] not only trivialise breast surgery, but also point to the apparent absurdity of a decision to allocate NHS funding to it, whilst at the same time objectifying (and with an accompanying picture, overtly sexualising) a woman’s breasts. Against this background, breast surgery has become an easy target for disinvestment [18].

However, evidence suggests that some breast surgery offers significant health gain [19, 20]. One local guideline summarises the evidence on breast reduction surgery:

*“Two systematic reviews and numerous primary studies conclude that breast reduction can reduce pain in shoulders, back and neck caused by large breasts. Patients generally express a high level of satisfaction following surgery. Other outcomes observed in research studies include improved psychosocial outcomes, psychological well-being, and quality of life. One cost effectiveness study calculated that the cost per quality-adjusted life year (QALY) for breast reduction was in the same range as that observed in hip replacement”* [21] *.*

This particular guideline then goes on to recommend that:

*“In the context of the resources available to provide healthcare for their populations, [the local commissioning organisations] consider the level of priority assigned to provision of breast reduction as low”* [21] *.*

At national level, the NHS Modernisation Agency (part of the English Department of Health from 2001–5) published guidelines for commissioners on when breast surgery should be NHS funded [22]. They recommend that female breast reduction be available if a patient is suffering from neck ache, backache and/or intertrigo, conditional that a professionally fitted bra has not relieved symptoms, and the patient’s body mass index (BMI) is under 30; that NHS surgery for gynaecomastia is allowable in post-pubertal patients with a normal BMI, and that breast enlargement be provided on an exceptional basis for women with an absence of breast tissue unilaterally or bilaterally, or with a significant degree of breast asymmetry.

A study of local implementation of these guidelines found that they were only followed in full by 7% of local commissioning organisations, with significant variation between them regarding which treatments were funded, eligibility criteria and specified thresholds. The authors concluded that: *“a ‘postcode lottery’ exists in the UK for plastic surgery procedures, despite national guidelines”* [23].

The lack of national data on IFRs means we have no clear picture of the variation in IFR decisions about breast surgery. In the locality quoted above, approximately a fifth of ‘plastics’ cases considered by the IFR panel were approved for funding in 2009/10 [16]. In another, none of the nine breast surgery cases considered over a three month period was approved [24]. There is additional variation in whether and how local commissioning organisations carry out pre-panel screening (‘triage’) of IFR referrals (undertaken by one or more individuals and led by either administrative or clinical staff), and the proportion of requests declined at this stage. In the two localities quoted above, a quarter and a third of all IFR cases triaged went to panel for review.

The wider context within which requests for NHS funding for breast surgery are considered is one of ambiguity and ambivalence about its status as a health care intervention (illustrated recently in the debate over whether the NHS should fund the replacement of faulty implants manufactured by Poly Implant Prothese (PIP) [25]). The clinical specialty of plastics typically draws a distinction between ‘reconstructive’ surgery (the correction of abnormality) and ‘cosmetic’ surgery (altering bodily appearance of patients presenting *“within the range of normality”*) [26]. Both are underpinned by aesthetic principles addressing bodily form and function, and mental and physical health. However, critics point out that the boundary between ‘normal’ and ‘abnormal’ is always contested, and that distinctions between reconstructive and cosmetic, form and function, psychological and physical are *“dubious and misleading, given their inherent interconnectedness”* [27]. Nonetheless, as Naugler points out, these definitions and distinctions *“have important ramifications for the acceptance, accessibility and personal meanings of specific plastic surgery procedures”* [27]. Other researchers have explored ‘cosmetic’ surgery’s precarious status between ‘beauty practice’, an ‘expression of identity’, and medical intervention in socio-cultural discourse [28, 29]. More prosaically, the word ‘cosmetic’ in everyday language is often used to convey superficiality, something *“without any real substance”* (Chambers English Dictionary).

Against this backdrop, our research explores how decisions about IFR breast surgery cases are made in practice. Studying deliberation illuminates how the notion of ‘value’ is dialogically constructed, and how ‘value’ judgements are made about who is deserving of NHS funding and who is not. Exploring how ‘deservingness’ is constructed in relation to health care becomes particularly salient at times of increasing pressure on NHS resources [30]. Although some researchers have explored ‘social values’ in health care rationing [31, 32], values are usually treated as pre-defined, fixed entities (for example ‘process’ values of transparency, accountability and participation, and ‘content’ values such as equity, solidarity and autonomy), that act as an ‘objective’ checklist to decision-making, rather than as contested, emergent and situated phenomena [33]. Our analysis aligns with those who view the process of ascribing value as an interpretive act involving ethical-moral choices, constructed moment-by-moment by social actors through interaction [34]. We take a practice-based view of deliberation, in which social life is explored as an “ongoing production” and decision-making a *“dynamic accomplishment rather than a static outcome”* [35].

Our research aims to illuminate how variation in health care (the ‘postcode lottery’) is produced locally through micro-rationing practices. Much of the research on health care variation explores this at a macro level of statistical analysis, seeking clues as to how to reduce ‘unwarranted variation’ [36]. But the question of how variation is constituted locally and how this intersects with the notion of value based commissioning remains unaddressed.

## Methods

The data presented here were part of a larger study of the rationing practices of IFR panels. The research received ethical approval from UCL Research Ethics Committee (ref: 0363/004), covering all sites involved. We undertook in-depth ethnographic exploration of the work practices of three IFR panels over a three-year period. Panels varied in their composition but typically included staff from the local commissioning organisation (directors of public health, nursing, finance and commissioning); a local general practitioner, pharmacist, and, in one site, a lay representative. In one locality (site A), nearly all panel discussions took place by email and we collected several thousand email exchanges; in the second site we audio-recorded one IFR panel discussion at which seven cases were discussed (site B); and in the third site we observed and took detailed field notes of five panel meetings covering a total of 15 cases (site C). In all three sites we interviewed panel members and collected associated documentation (evidence reviews, correspondence with referring clinicians and patients). Written informed consent was obtained from research participants. Our data set included 19 discussions (by email) about breast surgery in site A, one in site B and two in site C. For the purposes of the research presented in this paper, we initially looked at all 22 cases and then progressively focused in-depth on three case discussions, one from each study site, covering the three main breast procedures.

We drew on Yanow’s conceptual framework for interpretive policy analysis to analyse the enactment of low priority treatment policies on breast surgery. The role of the interpretivist policy analyst is to uncover the ‘architecture of policy arguments’ and *how* a policy means [37]. Yanow identifies three categories of ‘symbolic artefacts’ as significant carriers of meaning: language, policy objects and acts. We explored [a] the *language* used by actors in panel discussions and minutes of their meetings; [b] policy *objects* such as guidelines and policy statements, referral letters from clinicians, patient representations, and legal judgements; and [c] the act of categorisation.

For each of the selected cases, the three authors engaged in independent close readings of data transcripts, field notes, and policy guidance, and then in joint analysis sessions, exploring the meanings conveyed through language, objects and acts. We brought to our analysis a range of sensitising concepts from linguistic ethnography [38]. Firstly, the contribution of Bakhtin on the evaluative nature of language and the concept of ‘voice’ as ‘speaking consciousness’ in which particular values or viewpoints are enacted [39]. For Bakhtin, language is always a site of social struggle; it is not a neutral linguistic resource, but is *“already ‘overpopulated’ with other people’s voices and the social practices and contexts they invoke”* [39]. Every time we speak, we assimilate and appropriate the words of others and populate them with our own meaning, our own ‘evaluative accent’ in what Bakhtin refers to as the “ideological becoming of a human being” [39].

Secondly, our analysis was sensitised by theoretical work on the micro-level practices of categorisation in institutions [40, 41]. For example, institutional categories are invoked as ‘fixed entities’ to help organise and process people through institutions and often reveal what is taken for granted as common sense. At the same time categories are contested and discursively constituted in deliberation as participants oscillate between generalisation and particularisation of a case [42].

## Results and discussion

We begin this section with a summary of the three cases selected for in-depth analysis. Some details have been fictionalised to ensure anonymity and the names given to patients are pseudonyms. The first case is a referral of a 14 year-old boy (Jack) suffering from bilateral gynaecomastia. The local commissioning organisation has received letters from Jack’s GP and a specialist breast surgeon:

*“It was a pleasure to review this extremely bright young man in clinic today along with his father.*

*Jack has been suffering from bilateral gynaecomastia from the age of seven, which has been gradually and progressively enlarging in size. His father had similar problems as a young man. This cosmetic impediment is causing Jack significant psychological stress and he finds it extremely embarrassing to undress in front of his peers. He is otherwise physically well developed with secondary sexual characteristics consistent with his age and has a slim frame.*

*[…]*

*Given the volume of his gynaecomastia he would most certainly require re-position of his nipple areola regardless of whatever kind of surgery he has. There may be some merit in combining liposuction with a more definitive procedure later on to positively influence the eventual cosmetic. Despite his age surgery could be offered to him as the problem is quite clearly present and appears to be largely fatty, incompatible with his physical frame.” [Extract from the breast surgeon’s referral letter to the IFR panel]*

The case is discussed by email over several weeks, by a panel of commissioning managers, both clinical and non-clinical (see Table 1). After an initial delay in the case being reviewed, a telephone call from the patient’s father indicating that his *“son is being bullied at school because of his condition and so the dad is obviously irate and would like some information”* (email from IFR administrator to panel), prompts consideration of the case. The ensuing discussion between panel members focuses on attempts to categorise the case in terms of the commissioning organisation’s funding mechanisms and whether or not the procedure is considered cosmetic. A decision is taken not to fund.

**Table 1:** **Extract from IFR panel email discussion about Jack’s case of gynaecomastia (site A)**

From	To	Email text
Asst Director Public Health	Commissioning Manager	Please circulate my view, thanks. We would normally refuse to fund this as it’s a cosmetic procedure, however we need to be sure about the following points:
		• significant adverse effect on activities of daily living
		• significant disfigurement and I think should request further information on these, in particular the first one. I also think we should seek the [Assistant Director of Children’s Commissioning] views since this request is for a 14 year old child.
Chair of IFR Panel	Director of Commissioning cc Panel	Could you have a look at this, please. I’m not sure that it’s a contract exclusion and I wonder if it should be covered by PbR [Payment by Results].
		Breast reduction is normally something done to women with big breasts. The patient here is a young male with an unusual condition called gynaecomastia. This usually occurring in early adolescence and is a potential exception under the sector’s low priority treatments policy.
		If this were a contract exclusion then I’d say we could not fund it at present on affordability grounds, but I wonder if it could be classified as something under PbR and would not be considered to be a contract exclusion?
		Happy to discuss further.
Director of Commissioning	Chair of IFR Panel; cc Panel	I do not believe it is covered under PbR. I can find no reference to this under PbR. The consultant has also mentioned in his letter that it is cosmetic. We also have to bear in mind if [NHS hospital trust] carried out a procedure on a child they could apply a 78% uplift for the procedure from the adult tariff.
Chair of IFR Panel	Director of Commissioning; cc Panel	Thanks. If the consultant says that this is cosmetic and it’s not covered under PbR then we must decline to fund this on affordability grounds and because of the low priorities treatment policy (noting that gynaecomastia is a potential exceptional circumstance in this policy).
Assnt Dir. of Finance	Panel	I consider this cosmetic and therefore suggest that we do not fund this.
Director of Nursing	Panel	Although I appreciate that this is very problematic for the individual this particular request is as outlined by the consultant surgery for cosmetic reasons therefore I suggest that we do not fund this particular request.
Public health specialist	Panel	I agree not to fund this procedure for cosmetic reasons.

The second case concerns a 48 year-old woman (Brenda) whose request was identified on the IFR summary form as *“plastics for breast asymmetry”*. The patient has a heart condition that required her to have a device fitted in her chest some years ago. When this was fitted she also had bilateral breast augmentation to camouflage the device. Complications resulted in the removal of one breast implant, causing breast asymmetry. The case was initially turned down at triage, but after letters of complaint from the patient’s MP [Member of Parliament] and a private plastic surgeon, was referred to the IFR panel. The panel meets monthly, and is unusual in that patients are offered the option of giving a 10 minute representation of their request, although in this case the patient had chosen not to attend. The panel discussion focused on whether Brenda could be defined as ‘exceptional’ or not. After a lengthy discussion the decision was that Brenda was not exceptional and her request declined.

We reproduce an extract from research ethnographic field notes from observation of an IFR panel meeting in site C in which Brenda’s case of breast augmentation was discussed.

*“The IFR request was made in September 2011 by the patient’s hospital consultant where the original treatment occurred. It stated:*

*‘The patient’s quality of life would be greatly impacted upon [if the treatment was not approved]. The patient is currently on anti depressants and does not feel comfortable to leave the house or have a sexual relationship with her partner as she will not allow herself to be seen undressed. The patient has a long history of [mental health condition], having the surgery could avoid any further deterioration in the patient’s mental health’.*

*The request was turned down at triage one month later. The decision letter contained the following statement:*

*‘The XXX Referral and Treatment Criteria, state that breast augmentation/revision of breast implant procedures are not routinely funded [and]… psychological/psychiatric morbidity is not a criteria for funding aesthetic procedures. The IFR cannot take into account social, personal or emotional issues as we can only make decisions based on health needs. Therefore, while sympathetic to the fact that your patient may be disappointed, in accordance with IFR Policy, this application will not proceed to the IFR Panel and funding will not be provided’*.

*The agenda papers indicated that this decision letter prompted the patient to involve her MP. The PCT customer services officer wrote to the chair of the IFR panel, stating:*

‘*The MP has written on her behalf seeking to have a positive impact on the IFR team’s decision. In line with the NHS Complaints Procedure, I would be grateful if you could help me respond to his enquiry and either reconsider the decision or provide me with a more detailed explanation of the reason why her request for the re-insertion of the breast implant has been denied’.*

*The documentation also included a letter from a private plastic surgeon whom the patient consulted, stating that the patient is:*

*‘at her wits end due to the cessation of her NHS treatment at the end of last year”*. … *“My feeling as a plastic surgeon both privately and during the NHS full time is that she should have this done as she now has gross breast asymmetry which makes garment wearing problematic. Therefore there is a functional as well as a psychological benefit to this operation. She was also halfway through her treatment that was allowed on the NHS up until the middle of last year and I think it is unfair that it was stopped before finalisation. In addition, her heart condition means that surgery elsewhere is prohibitively dangerous as she needs cardiology and cardiothoracics to be present as well as a cardiac anaesthetist. This means I cannot do the surgery for her here. I think [the hospital] would be the most appropriate place for her to have this re-insertion of an implant’.*

*The Chair (Director of Nursing) summarised the above case history, noting the patient’s high anaesthetic risk. She asked the panel if they thought this patient was exceptional? The Director of Commissioning said that he didn’t see the patient as exceptional, although the circumstances leading to the request ARE exceptional, what she’s asking to have done ISN’T.*

*[…]*

*The public health consultant said that from the panel’s viewpoint they’re most concerned about the FUNCTIONAL benefits of a procedure. He quoted from the private consultant’s letter:*

*‘garment wearing [is] problematic. Therefore there is a functional as well a psychological benefit….’*

*There was agreement that this is not functional benefit in the sense that the panel defines it. The chair said that functional benefit has to be clinical benefit.*

*The GP member then commented:*

*‘it’s a difficult one, how do we rationalise patients having implants after breast cancer surgery [the policy explicitly says it does not apply to patients undergoing breast reconstruction as part of treatment for breast cancer] but not for an infection such as this? [the implant had apparently originally been removed because of an infection and complications]. “There’s a bit of me saying what’s the difference?’*

*[….]*

*The pharmacist suggested that the IFR request ‘boils down to an application for making someone symmetric who is asymmetric, so at the end of the day it’s a cosmetic procedure’.*

*The Director of Commissioning (who at the start had said he did not believe the patient was exceptional) seemed to agree with the GP that ‘if we do it for cancer patients then perhaps we should do it for this patient who genuinely needed the operation to have the [device] in the first place’.*

*At this point the panel were struggling and fumbling, clearly not at all sure where to take the discussion next. The GP, an authoritative member of the panel, had made a strong case for this patient to be treated as exceptional in the same way as the policy treats cancer patients as exceptional. At the same time, the referring clinician had not made a strong case for clinical benefit (within the narrow definition of clinical benefit being used by the panel) and there was a sense in which this was ‘at the end of the day’ cosmetic surgery, which the policy clearly says the PCT won’t pay for.*

*The GP repeated his views about personally not seeing the difference between this patient and cancer patients. The Director of Commissioning agreed with him, saying his view was the same as the GP’s.*

*[… ]*

*The Director of Commissioning (trying to bring the panel to some decision I sensed) said I don’t think this meets exceptionality in the broadest sense (now returning to his starting position). The chair asked each panel member what they thought:*

*Pharmacist – ‘not exceptional’*

*Lay member – ‘not exceptional’*

*Chair – ‘not exceptional’*

*GP – ‘I think it is’.*

*Chair – ‘I’m sorry GP you’re outnumbered on this one. Is that OK?’*

*GP – ‘Yes, it’s OK’.*

The third case concerns a 38 year-old woman, Jane (see Table 2). Her GP reports that she is suffering from backache due to her large breasts and that *“the patient states physiotherapy and analgesia have not helped”*. The documents considered include a letter from the patient to her GP putting forward her case for surgery, and a report from the local physiotherapy department, noting that the patient responded *“partially to the rehabilitation program and has been discharged”*. The case is considered by a panel that meet weekly. They discuss the details of the physiotherapist’s report and patient’s letter and reach agreement that the case is exceptional and funding is approved subject to confirmation of the patient’s BMI.

**Table 2:** **Extract from transcript of audio-recording of IFR panel meeting about Jane’s case of breast reduction (site B)**

Speaker	Spoken words
Chair	Moving on to this case which is breast reduction, for back ache, we haven’t really… (inaudible)
CPH	How old is she? (pause whilst members look through papers including a report from a physiotherapist and a letter from the patient in support of her application)
ADPH	The physio report, doesn’t actually recommend, or what it says is that patient has responded partially [to
Chair	partially]
ADPH	the rehab programme and has been discharged.
Chair	mmm
ADPH	she needs a good bra
GP	I mean I think her letter actually says very much more [than
ADPH	yes] exactly
GP	anything, now it’s a question of whether, you know, she’s not had a relationship, she feels embarrassed, there’s obviously a psychological=
ADPH	hardly (inaudible) 38DD=
GP	=well if she is 4 foot 11 and quite a petite [frame
ADPH	what’s her] BMI?
GP	then that could well be large. And the fact that she has had three children will make them much more pendulous anyway so I could accept that she says they hang to her stomach and all the rest of it.
Chair:	I think the main thing here is that she hasn’t been referred, she doesn’t appear to have been referred to an orthopedic surgeon [or
GP	breast] reduction, well
Chair	to get a second clinical opinion. We’ve got the GP’s opinion but she hasn’t had any MRIs or anything like that. We don’t know what the other potential factors could be.
GP	I don’t know that an MRI would help to be honest. I think there are much more psychological issues with this lady than (.) the backache is to be honest neither here nor there. I am actually more concerned about the fact that she is withdrawing, she can’t pick up her child, she feels embarrassed to have an intimate relationship, umm
ADPH	She’s got a very young child, a 2 year old
GP	She’s got very young children, and I think [that’s
ADPH	yeah]
GP	that’s making her exceptional personally and I would say, I would approve then
Pharmacist	(inaudible)
GP	I mean the only thing I could check is what her BMI is=
ADPH	=I think that’s the only thing I wanted
GP	whether there is a, you know, if she was a BMI of 40 then maybe losing=
Pharmacist	weight
GP	weight would actually make them less heavy but she might still need a procedure to make [them
ADPH	I think] in principle we would agree but I mean I also want to know what her BMI is though

GP = general practitioner. ADPH = Assistant Director of Public Health. CPH = Consultant in Public Health.

Transcribing conventions: = no pause between speakers; [ onset of overlapping speech; ] end of overlapping talk.

Below, we present a micro-analysis of these cases to show how, through the artefacts of symbolic language, objects and acts, meaning is constructed and communicated, and IFR panels create their own ‘interpretive communities’ within which local decisions are justified. For the purpose of this analysis we put to the background the different modes of communication (email, face-to-face); panel composition (for example the inclusion of lay representatives), and the particular local financial contexts. A more in-depth account of these and other aspects of IFR decision-making can be found elsewhere [43–45].

### Symbolic language

A notable feature of the presentation and discussion of Jack’s case is the evaluative function of the word ‘cosmetic’. According to Bakhtin, all language is inherently evaluative and passes judgement on the world as it describes it [39]. In the referral letter, the consultant builds up a strong case of exceptionality (in line with the requirements of IFR decision-making as described above), referring to the boy’s “*suffering*”, *“significant psychological distress”, “extremely embarrassing”, “the volume of his gynaecomastia”*, all phrases that emphasise the validity of his exceptional status. Within the context of this description of ‘exceptionality’, the reference to the *“cosmetic impediment”* arguably emphasises the impediment rather than its ‘cosmetic’ nature. The dissonance between the two words is striking: an impediment is not merely ‘cosmetic’. The consultant describes his treatment plan *“to positively influence the eventual cosmetic”*; the word ‘cosmetic’ here suggests a medical textbook meaning, i.e. surgery altering the appearance of the body.

However, in the email discussion the panel gives a very different evaluative accent to the word cosmetic. Here ‘cosmetic’ references a category of treatment in their low priority treatment policy that they *“normally refuse to fund”*. By labelling the request ‘cosmetic’, the panel indexes a common sense institutional classification system that, in the words of Mary Douglas *“describes the way things are”* [41], and the request easily loses its entitlement to funding. Moreover, at the same time as panel members give the word a new evaluative accent, they appropriate the surgeon’s voice, saying “*The consultant has also mentioned in his letter that it is cosmetic”*, and *“If the consultant says that this is cosmetic… then we must decline to fund…”*. In other words, they attribute *their* (the panel’s) evaluative accent to the *surgeon’s* voice, introducing ‘attributional distance’ [46] between themselves and their decision not to fund.

This example shows how the different evaluative accent of a single word functions to override the considerable work the consultant does to build a case for funding. Following Bakhtin, we suggest that the evaluative accent the panel gives to the word ‘cosmetic’ is not only a reflection of the here-and-now of policy discourse, but also contains echoes of *“historical and cultural scripts”* [42], invoking societal views of cosmetic surgery more as ‘beauty practice’ than ‘medical intervention’ [28], and thus as something inherently undeserving of NHS funds.

The data extracts contain other instances of the evaluative accenting assigned to specific words and phrases. The word ‘functional’ for example, acquires a number of meanings in relation to breasts. The breast functions as an organ of lactation and a secondary sexual characteristic as well as (pathologically) interfering with the functions of other organs and structures (e.g. the pectoral muscles and thoracic spine). As a surface structure, it also functions more symbolically as part of a healthy body contour and plays a significant role in gender identity.

In Brenda’s case, the consultant makes a case that *“there is a functional as well as psychological benefit to this operation”* because *“she [the patient] now has gross breast asymmetry which makes garment wearing problematic”* – a comment that explicitly emphasises the breast’s abnormal *biomechanical* function while also acknowledging its symbolic one in shaping body image and identity*.* However, the panel fails to engage with this definition of ‘functional’, stating that functional benefit has to be *“clinical benefit”.*

In fact, although significant functional impairment (and the potential to reverse it) is sometimes identified as a relevant criterion for breast *reduction*, we found no guidelines referring to it for breast augmentation (for example to correct gross asymmetry). For breast reduction surgery, some commissioning organisations specified what they meant by significant functional impairment, but there was no consistency across organisations. In one locality, for example, the emphasis was on *social* function: *“Symptoms prevent the patient fulfilling vital work or educational responsibilities, or symptoms prevent the patient carrying out vital domestic or carer activities”.* More commonly (as in Jane’s case below), guidelines defined functional impairment in terms of physical function, such as back or shoulder pain.

These examples of symbolic language illustrate how, in making their case for funding, doctors use particular words, either because they are part of their everyday medical lexicon, or perhaps because they are aware of the rules which govern funding policies. However, each locality has different rules (and doctors may deal with several commissioning organisations), and the rules are differently drawn upon and interpreted within IFR panel deliberations. Each decision is therefore contingent on how words come to acquire specific meanings in practice.

### Symbolic objects

Taking Yanow’s second category of policy artefacts, we explore the role of documents as symbolic objects carrying meaning. A significant proportion of IFR panel activity involves interpreting and making sense of cases through considering documents - policy guidelines, referral letters, patient letters, and so on. Typically, a collection of papers relating to each case is circulated to panel members before the meeting (or as part of an email discussion). At the discussions we observed members spent time shuffling papers, looking back and forth between papers, and reading aloud from documents to draw the panel’s attention to specific extracts. As previous research has shown, documents formed a critical part of the groups’ sense-making activities, comprising *“articulating, debating and validating different readings”* of documents [47], as they created a shared narrative of the case [48]. In this section we explore the work that documents achieve in foregrounding particular concerns. Specifically, we explore how documents inscribe both institutional and patient framings of a funding request, creating a ‘dilemma of attention’ for panel members, as they struggle to attend to the patient’s concerns on the one hand and institutional concerns on the other [49].

In Jack’s case, the consultant’s referral letter emphasised the psychological aspects of the patient’s condition (although these were not discussed by the panel). Across our dataset we noted that this emphasis on psychological factors was common in referral letters for breast surgery. We also noted the following patient information on the NHS Choices website: *“Cosmetic surgery is rarely available through the NHS. There must be a major physical or psychological reason for needing the surgery”*. We might assume that this guidance encourages patients and their doctors to emphasise psychological factors in arguing their case.

However, in Brenda’s case, reference to psychological morbidity becomes a reason for *refusing* a funding request for funding. The initial ‘triage stage’ decision letter, in which the patient’s doctor is informed that *“this application will not proceed to the IFR panel and funding will not be provided”,* quotes from the local policy document, stating that *“psychological/psychiatric morbidity is not a criteria for funding aesthetic procedures”* (incidentally, a clause that was added to the 2011 version of the policy document). The decision letter goes on to say: *“the IFR cannot take into account social, personal or emotional issues as we can only make decisions based on health needs”*. This latter sentence refers to a widely cited but erroneous interpretation of case law, which we have discussed previously [44]. As we set out in that paper, a number of recent legal judgements have confirmed that when considering IFR cases, commissioning organisations do not *have* to take account of ‘social factors’ in their assessment of exceptional circumstances, but this legal position is frequently interpreted, as here, as meaning that they *cannot* take social factors into account. The overall effect of the decision letter containing these policy quotes is to suggest that reference to psychological factors in the request ‘ruled out’ its consideration as an IFR, and furthermore, the suggestion is that psychological morbidity, however profound, is not a ‘health need’.

This example shows how the local policy document is crucial in defining meaning. Reference to it serves to emphasise an institutional framing of the IFR and remove the need for deliberating the merits of the specific case at a panel meeting; it becomes a more or less straightforward case of ‘the policy says no’. In our interviews with panel members, respondents expressed concern about the increasingly significant role of pre-panel triage in the IFR process. They suggested that important perspectives (those of the patient, GP, or lay representative), widely recognised as essential to fair deliberation of complex cases [50, 51], are being marginalised from IFR decision-making, and superseded by an algorithmic, technocratic approach to potentially ‘exceptional’ cases, which by their very definition, should not be judged against a set of rules.

Policy guidance is only one type of IFR documentation. Additionally, there are referral and advocacy letters setting out the patient’s case, and whether, how and to what extent these are brought into and able to influence discussions. In Brenda’s case, after the initial rejection of the IFR at triage, letters from the patient’s MP and a private plastic surgeon bring further authoritative voices to the table and prompt consideration of Brenda’s case by the IFR panel, making it possible that the patient’s framing of the case can take a hold (see next section). However, ultimately it is the institutional framing of exceptionality that dominates discussion and leads to a decision not to fund. Similarly, in Jack’s case the patient’s voice is conveyed through the consultant’s referral letter and a phone call from the father, but in the ensuing email discussion it quickly evaporates and attention focuses on institutional concerns.

By contrast, in Jane’s case the patient’s voice is successfully privileged over the words in the policy documents by the GP framing the discussion in the patient’s terms early on. The GP responds to another member who is summarising the physiotherapist’s report, saying *“I think [the patient’s] letter actually says very much more than anything…”*. The patient’s condition and circumstances quickly become the focus of attention, with the GP animating the patient’s own words (*“she can’t pick up her child, she feels embarrassed to have an intimate relationship…”*) [52]. The rhetorical work of the GP’s contribution is apparent: she highlights the patient’s role as a mother of three children, and she prefaces her animation of the patient’s words by emphasising her concerns about *“the fact that [the patient] is withdrawing”*, adding her professional legitimacy to the patient’s voice.

Interestingly, in Jane’s case, far from being a reason NOT to fund, psychological morbidity is the explicit reason *for* the panel to fund the request. Even though the local policy states that: *“Funding should be considered if there are clear physical problems e.g. serious functional impairment, significant neck/back pain, intertrigo AND BMI < 30”*, the GP argues that *“there are much more psychological issues with this lady than*.., *the backache is to be honest neither here nor there”,* and successfully persuades the panel to agree in principle to fund Jane (once she has checked Jane’s BMI with her GP).

Again, our analysis of how these artefacts are drawn upon in the discussion highlights how highly contingent the course of deliberation and decision-making can be. Which particular phrases or words from a policy are quoted, how they are interpreted, the different ‘voices’, in the Bakhtinian sense, that are present and brought into and frame discussion, all contribute to how panels create their own specific interpretation and narrative of the case, that leads them to a decision to fund or not.

A striking feature of all three cases was the *absence* of certain documents from discussion. Whereas a characteristic of many IFR cases was the sizeable amount of research evidence (literature reviews and/or original research papers) included as background documentation and brought into discussion, with breast surgery cases there was a surprising lack of reference to formal evidence. This contributes to the sense that breast cases were not taken as seriously as some other requests and did not warrant the attention to scientific research. This was also reflected in some of the ‘common sense’ assumptions made about these requests. For example, our field notes record the chair of an IFR panel introducing one case to the panel with the comment *“Today’s breast augmentation! Do we ever have a week when we don’t have one!”*, and another IFR panel member commenting in an interview that *“with the beauty interventions, we know to decline them, … we don’t really think about it”.* Arguably, these comments reflect and reproduce deeply ingrained cultural views about the triviality and even comical nature of breasts and cosmetic surgery, what we might refer to as the ‘boob-job’ perspective.

### Symbolic acts

Yanow’s third category of artefacts are ‘symbolic acts’. Here we focus on the act of categorising, as part of the process of deciding what to fund. Categorising is a fundamental part of institutional work, it legitimates what institutions do by describing the way things are in the social world [41, 53], it enables institutions to ‘sort things out’ [54], and makes possible the people-processing activities in which institutions are engaged [42]. Crucially, these authors emphasise the act of categorising as involving moral evaluation; in the case of IFRs, deciding whether a patient is deserving of NHS funds.

Individual funding requests involve an enormous amount of categorising – panel members deliberate about whether a patient’s case fits the IFR category or not, fits the category of exceptionality or not, is about psychological morbidity or not, and so on. The act of categorising may be quite obvious, or it may be more subtle and concealed. In Jack’s case, for example, the chair of the panel comments:

*“Breast reduction is normally something done to women with big breasts. The patient here is a young male with an unusual condition called gynaecomastia. This usually occurring [sic] in early adolescence and is a potential exception under the sector’s low priority treatments policy.”*

This statement not only places Jack in a diagnostic category that is a *“potential exception”* under local policy, but also serves to set apart the category of *“women with big breasts”* applying for breast reduction from those with the *“unusual condition”* of gynaecomastia, emphasising the exceptionality (and arguably deservingness) of (young) male cases of breast surgery. In the end, however, as we argued above, the labeling of Jack’s request as ‘cosmetic’ trumps all other arguments, and his ‘exceptionality’ fails.

In Brenda’s case the GP on the panel questions the categorisation of patients as set out in local policy. He says that he cannot really see the difference between patients having implants after breast cancer surgery (which the NHS automatically funds) and this patient who is requesting re-insertion of an implant after clinical complications related to her heart condition. But later in the discussion, the community pharmacist member suggests that *“the request boils down to an application for making someone symmetric who is asymmetric, so at the end of the day it’s a cosmetic procedure”,* and eventually, after considerable discussion, the GP is outvoted and the request refused.

The pharmacist’s intervention is an example of what scholars of rhetoric refer to as argument by association [55]. By dissociating asymmetry from the ‘deserving’ category of cancer patients, and associating it with the implicitly-agreed-to-be ‘undeserving’ category of a ‘cosmetic procedure’, ‘boiling down’ the case to one of asymmetry, the request easily fits the organisation’s policy not to fund (breast augmentation *“is not routinely funded within the local NHS for any patient group”*). Incidentally, a year earlier, the local policy *did* permit breast augmentation for gross asymmetry. The basis for this change in policy wording is unclear, especially given national guidelines stating that *“exception should be made for women with a significant degree of asymmetry of breast shape and/or volume”* [22].

## Conclusion

Current health policy debates emphasise the need to tackle ‘unwarranted variation’ in access to NHS treatments, including those on ‘low priority’ lists, by ensuring uniformity of local guidelines and policies [6, 12]. Such recommendations have an intuitive appeal. It is undoubtedly difficult to understand why in one locality breast augmentation may be eligible for NHS funding to correct asymmetry if *“there is a disparity of 2 or more cup sizes in the lower range (size C or below) or 3 or more cup sizes in the upper ranges (size D upwards)”*, but in another locality the same procedure is ineligible for NHS funding for any patient group. Or why in one locality *“significant psychological problems due to poor body image” is* permissible as an exceptional circumstance for breast reduction surgery, whereas in another locality *“psychological morbidity is not a criterion”* for funding it. Such ‘unwarranted variation’ lends strong support to the recent recommendation from the British Medical Association that commissioning organisations should collaborate to ensure consistent policies across localities, with greater reference to national guidelines [6].

However, our findings suggest that ultimately the search for uniformity is likely to be an illusory policy goal. There exists a fundamental paradox at the heart of the IFR system for rationing health care resources. On the one hand it is a system predicated on and requiring case-based reasoning and judgement, to ensure that funding decisions are based upon individual patient circumstances [15]. On the other, it is a system that has evolved to become increasingly formalised and bureaucratised, with more and more emphasis on the use of pre-defined rules (eligibility criteria, attempts to define exceptionality, treatment thresholds, and so on). In our study we observed a discernible tension for panel members between framing cases as complex instances of human suffering, involving moral engagement and emotionally and clinically challenging judgements, and instances of technocratic processing, involving instrumental negotiations over policy fit.

The turn to technocratic and rule-based reasoning is understandable in that it makes manageable the difficult tasks of rationing health care and critically appraising the clinical judgement of referring clinicians. The privileging of ‘system’ rules allows panel members to sidestep the fundamental ethical choices at the heart of IFR cases. But – and this is the real illusion of a standards based reasoning approach – rules must always be interpreted [56]. Our findings suggest that, even if greater uniformity of local policies were achieved, each local decision-making group will still create their own interpretive community within which local decisions will be justified. The subtleties of social interaction ensure that a putatively ‘rational’ system for decision-making remains a largely idiosyncratic one.

What are we to make of the shift in terminology from referring to procedures such as breast surgery as ‘low priority treatments’ or ‘procedures of limited clinical effectiveness’ to ‘value based commissioning’? Certainly this sort of linguistic work invokes a more positive image; it would be hard to disagree with the notion of commissioning being based on some sort of value. And the similarity of the term with ‘values based commissioning’, which explicitly privileges patient defined needs and values in the commissioning of mental health services [57], is perhaps not coincidental. The problem, however, is that accounts of value based commissioning present ‘value’ as an incontrovertible ‘fact’ that requires no further explanation, and can even be measured and audited [8]. In contrast, we have demonstrated how, in relation to individual cases, value is essentially indeterminate and uniquely constituted in situated contexts through dialogical reasoning. Despite the rhetoric of value based commissioning, and the evidence of clinical benefit of some breast surgery, our study indicates that breast surgery’s precarious status as a legitimate health care intervention in the NHS to alleviate pain and suffering is likely to remain.

The implications of our findings extend beyond breast surgery and IFRs in the NHS. By looking at instances of decision-making close up, we have illuminated how health care rationing policy and practices, as interpretive acts, inevitably involve judgements of moral worth and deservingness, and can never be simply evidence-based endeavours. In this sense, our findings contribute to a growing body of research [58–62] that fundamentally challenges the scientistic thinking [34] underpinning mainstream health policy discourse.

## Endnotes

^a^NHS commissioning organisations are responsible for commissioning health services for their local population. They control around two thirds of the NHS budget. At the time of this study 152 ‘primary care trusts’ (PCTs) commissioned local health services in England; in April 2013 PCTs were replaced with approximately 200 clinical commissioning groups (CCGs).

^b^Commissioning organisations delegate decisions on requests from patients and their doctors to fund a particular test or treatment as a ‘one-off’, even though the NHS (at least in that locality) does not normally fund it to ‘individual funding request’ (IFR) panels. Panels vary in their composition from locality to locality but typically include commissioning organisation staff (e.g. directors of public health, finance, nursing), local general practitioner(s), a pharmacist, and sometimes lay representatives.

## Abbreviations

BMI: Body mass index
IFR: Individual funding request
NICE: National Institute of Health and Clinical Excellence
NIHR: National Institute for Health Research
PIP: Poly Implant Prothese
QALY: Quality adjusted life year.

## References

[1] Adams S: *Third of Health Authorities ‘Still Imposing Blanket Ban Treatments’*. http://www.telegraph.co.uk/health/healthnews/9539341/Third-of-health-authorities-still-imposing-blanket-treatment-bans.html Telegraph. 12 Sep 2012

[2] Department of Health (2011). The Operating Framework for the NHS in England 2012/13.

[3] National Audit Office (2012). Progress in Making NHS Efficiency Savings.

[4] Commission A (2011). Reducing Spending on Low Clinical Value Treatments.

[5] Rumbold B, Alakeson V, Smith P (2012). Rationing Health Care. Is it Time to Set out More Clearly what is Funded by the NHS?.

[6] Al-Zaidy S (2013). National Versus Local Equity. How much Variation is Acceptable to Doctors?.

[7] NHS North Central London (2012). Policy for Procedures of Limited Clinical Effectiveness.

[8] Gray M, Swift S, Suleman M, Hakes D, Qualie M, Mitchell A, Dutch S, Beasley N (2012). Value Based Clinical Commissioning of Elective Surgical Care.

[9] Coronini-Cronberg S, Lee H, Darzi A, Smith P (2012). Evaluation of clinical threshold policies for cataract surgery among English commissioners. J Health Serv Res Policy.

[10] Federation of Surgical Speciality Associations: *Open letter from the Federation of Surgical Specialty Associations*. http://www.guardian.co.uk/society/2011/apr/18/open-letter-federation-surgical: Guardian 11th May; 2011

[11] Garner S, Littlejohns P (2011). Disinvestment from low value clinical interventions: NICEly done?. BMJ.

[12] Maybin J, Klein R (2012). Thinking about Rationing.

[13] Austin D (2008). Priority Setting: Managing Individual Funding Requests.

[14] National Prescribing Centre (2009). Supporting Rational Local Decision-Making about Medicines (and Treatments). A Handbook of Good Practice Guidance.

[15] Keogh B (2011). Access to Services. Letter to SHA Medical Directors 21/09/11.

[16] Healthcare Priorities Unit (2010). Annual Report on Individual Funding Requests 2009–10.

[17] **Why did NHS Pay for this Woman's 36DD Breasts but Refuse to pay £24,000 for an Operation so this Boy can Walk?** [http://www.dailymail.co.uk/news/article-2350581/Why-did-NHS-pay-womans-36DD-breasts-refuse-pay-operation-boy-walk.html]

[18] Robinson S, Dickinson H, Freeman T, Williams I (2011). Disinvestment in health - the challenges facing general practitioner (GP) commissioners. Public Money Manag.

[19] Breuning EE, Oikonomou D, Singh P, Rai JK, Mendonca DA (2010). Cosmetic surgery in the NHS: Applying local and national guidelines. J Plast Reconstr Aesthet Surg.

[20] Klassen A, Fitzpatrick R, Jenkinson C, Goodacre T (1996). Should breast reduction surgery be rationed? A comparison of the health status of patients before and after treatment: Postal questionnaire survey. BMJ.

[21] South East Coast Policy Recommendation Committee (2011). Policy Recommendation: Breast Reduction.

[22] NHS Modernisation Agency (2005). Information for Commissioners of Plastic Surgery. Referrals and Guidelines in Plastic Surgery.

[23] Henderson J (2009). The plastic surgery postcode lottery in England. Int J Surg.

[24] Korhonen T (2011). Individual Funding Request Panel Quarterly Report to NHS Swindon Board.

[25] Smith R: **NHS Bill for PIP Breast Implant Scandal is Almost £2m so Far.** [http://www.telegraph.co.uk/health/healthnews/9605030/NHS-bill-for-PIP-breast-implant-scandal-is-almost-2m-so-far.html]

[26] Burd A (2008). Plastic, reconstructive and aesthetic surgery. Med Bull.

[27] Naugler D, Heyes CJ, Jones M (2009). Crossing the Cosmetic/Reconstructive Divide: The Instructive Situation of Breast Reduction Surgery. Cosmetic Surgery: A Feminist Primer.

[28] Gimlin D (2000). Cosmetic surgery: Beauty as commodity. Qual Sociol.

[29] Gimlin D (2007). Accounting for cosmetic surgery in the USA and Great Britain: a cross-cultural analysis of women's narratives. Body Soc.

[30] Griffiths L, Hughes D (1994). ‘Innocent parties’ and ‘disheartening’. Experiences: Natural Rhetorics in Neuro-Rehabilitation Admissions Conferences. Qual Health Res.

[31] Clark S, Weale A (2012). Social values in health priority setting: a conceptual framework. J Health Org Manag.

[32] Littlejohns P, Sharma T, Jeong K (2012). Social values and health priority setting in England: “values” based decision making. J Health Organ Manag.

[33] Stone D, Morone J, Litman T, Robins S (2008). Values In Health Policy: Understanding Fairness and Efficiency. Health Politics and Policy.

[34] Miller C, Simons H (1990). The Rhetoric of Decision Science or Herbert A. Simon Says. The Rhetorical Turn. Invention and Persuasion in the Conduct of Inquiry.

[35] Feldman MS, Orlikowski WJ (2011). Theorizing practice and practicing theory. Organ Sci.

[36] Tanenbaum SJ (2013). Reducing variation in Health Care: The Rhetorical Politics of a Policy Idea. J Health Polit Policy Law.

[37] Yanow D (2000). Conducting interpretive policy analysis.

[38] Maybin J, Tusting K, Simpson J (2011). Linguistic Ethnography. Handbook of Applied Linguistics.

[39] Maybin J, Wetherall M, Taylor S (2001). Language, Struggle and Voice: The Bakhtin/Vološinov Writings. Discourse Theory and Practice.

[40] Billig M (1987). Arguing and Thinking: A Rhetorical Approach to Social Psychology.

[41] Douglas M (1986). How Institutions Think.

[42] Mäkitalo (2003). Accounting practices as situated knowing: Dilemmas and dynamics in institutional categorization. Discourse Stud.

[43] Russell J, Greenhalgh T, Burnett A, Montgomery J (2011). No decisions about us without us? Individual healthcare rationing in a fiscal ice age. BMJ.

[44] Russell J, Greenhalgh T (2012). Affordability as a discursive accomplishment in a changing National Health Service. Soc Sci Med.

[45] Russell J, Greenhalgh T (2013). Being rational and being human: How National Health Service rationing decisions are constructed as rational by resource allocation panels. Health.

[46] Clayman SE, Drew P, Heritage J (1992). Footing in the Achievement of Neutrality: The Case of News-Interview Discourse. Talk at Work: Interaction in Institutional Settings.

[47] Freeman R, Maybin J (2011). Documents, practices and policy. Evid Policy.

[48] Gkeredakis E, Swan J, Nicolini D, Scarbrough H (2011). Rational Judgement Revisited: Practices of Deliberation in Healthcare Funding Decisions.

[49] Swinglehurst D, Greenhalgh T, Russell J, Myall M (2011). Receptionist input to quality and safety in repeat prescribing in UK general practice: ethnographic case study. BMJ.

[50] Daniels N (2000). Accountability for reasonableness. BMJ.

[51] Klein R (2005). A middle way for rationing healthcare resources. BMJ.

[52] Goffman E (1981). Footing. Forms of Talk.

[53] Stone D (1997). Policy Paradox. The Art of Political Decision Making.

[54] Bowker GC, Star SL (2000). Sorting Things Out: Classification and its Consequences.

[55] Perelman C, Olbrechts-Tyteca L (1971). The New Rhetoric: A Treatise on Argumentation.

[56] Hunter KM (1989). A science of individuals: Medicine and casuistry. J Med Philos.

[57] Dent E (2013). ‘Values based’ commissioning aims to put users’ views at the heart of reshaping services. Health Serv J Supp.

[58] Stone DA (1984). The Disabled State.

[59] Wood M, Ferlie E, Fitzgerald L (1998). Achieving clinical behaviour change: a case of becoming indeterminate. Soc Sci Med.

[60] Green J (2000). Epistemology, evidence and experience: evidence based health care in the work of Accident Alliances. Sociol Health Illness.

[61] Gabbay J, le May A, Jefferson H, Webb D, Lovelock R, Powell J, Lathlean J (2003). A case study of knowledge management in multi-agency consumer informed ‘communities of practice’: implications for evidence-based policy development in health and social services. Health.

[62] Jenkings KN, Barber N (2004). What constitutes evidence in hospital new drug decision-making?. Soc Sci Med.

[CR63] The pre-publication history for this paper can be accessed here:http://www.biomedcentral.com/1472-6963/14/413/prepub

